# Effect of feeding mode on infant growth and cognitive function: study protocol of the Chilean infant Nutrition randomized controlled Trial (ChiNuT)

**DOI:** 10.1186/s12887-020-02087-9

**Published:** 2020-05-18

**Authors:** Rosario Toro-Campos, Cecilia Algarín, Patricio Peirano, Marcela Peña, Teresa Murguia-Peniche, Steven S. Wu, Ricardo Uauy

**Affiliations:** 1 Institute of Nutrition and Food Technology (INTA), University of Chile, Av El Libano 5524, Macul, Santiago, Chile; 2 Psychology Department, Pontific Catholic University, Santiago, Chile; 3 Medical Affairs, Mead Johnson Nutrition, Evansville, IN USA

**Keywords:** Infant formula, Milk fat globule membrane, MFGM, growth and development, Clinical trials, Breast feeding, Chile

## Abstract

**Background:**

A central aim for pediatric nutrition is to develop infant formula compositionally closer to human milk. Milk fat globule membranes (MFGM) have shown to have functional components that are found in human milk, suggesting that addition of bovine sources of MFGM (bMFGM) to infant formula may promote beneficial outcomes potentially helping to narrow the gap between infants who receive human breast milk or infant formula. The objective of the current study is to determine how the addition of bMFGM in infant formula and consumption in early infancy affects physical growth and brain development when compared to infants fed with a standard formula and a reference group of infants fed with mother’s own milk.

**Methods:**

Single center, double-blind, and parallel randomized controlled trial. Planned participant enrollment includes: infants exclusively receiving breast milk (*n* = 200; human milk reference group; HM) and infants whose mothers chose to initiate exclusive infant formula feeding before 4 months of age (*n* = 340). The latter were randomized to receive one of two study formulas until 12 months of age: 1) cow’s milk based infant formula that had docosahexaenoic (DHA) (17 mg/100 kcal) and arachidonic acid (ARA) (25 mg/100 kcal); 1.9 g protein/100 kcal; 1.2 mg Fe/100 kcal (Standard formula; SF) or 2) a similar infant formula with an added source of bovine MFGM (whey protein-lipid concentrate (Experimental formula; EF). Primary outcomes will be: 1) Physical growth (Body weight, length, and head circumference) at 730 days of age; and 2) Cognitive development (Auditory Event-Related Potential) at 730 days of age. Data will be analyzed for all participants allocated to each study feeding group.

**Discussion:**

The results of this study will complement the knowledge regarding addition of bMFGM in infant formula including support of healthy growth and improvement of neurodevelopmental outcomes.

**Trial registration:**

NCT02626143, registered on December 10th 2015.

## Background

Multiple observational and experimental studies support the idea of a “sensitive period” or “window of opportunity” to achieve optimal physical growth and cognitive development [1]. The first 1000 days (from conception to 2 years postnatal) are considered as a critical period and lay the foundations for a health and well-being across the human life span [2]. Thus, provision of adequate nutrition and psychosocial stimulation during this window supports typical cognitive development, linear growth and healthy metabolism in ways that may decrease risk for long-term childhood- or adult-onset chronic disease [3].

For infants, human milk feeding is recognized as the gold standard for infant nutrition. In addition to supporting healthy growth and development [4, 5], breastfed vs. non-breastfed infants have lower risks of childhood obesity, type 2 diabetes, and a protective effect against elevated systolic blood pressure, and a higher performance on cognitive development measures [6]. Therefore, a central aim of pediatric nutritional research, which in turn has guided the development of infant formula to bring it compositionally closer to human milk, is to better meet the needs of formula-fed infants.

One example is the evolution of expert recommendations for the iron content of infant formula [7–10]. Furthermore, ongoing research has highlighted the importance of previously less recognized functional components present in human milk, including complex lipids, proteins, and oligosaccharides [11]. A number of these functional nutrients are present within milk fat globular membrane (MFGM), the protein-rich phospholipid membrane structure that surrounds milk fat droplets as they are naturally secreted. MFGM consists of a triple-layer phospholipid membrane containing a variety of other bioactive lipids as well as integral proteins and glycoproteins [12]. MFGM components, including gangliosides, sphingomyelins, sialic acid, choline and cholesterols, have demonstrated beneficial biological effects in preclinical animal models, including improved learning ability as well as myelination in rodents [13–15] and grey and white matter brain growth and spatial learning in piglets [16]. Preclinical studies in adult humans have demonstrated effects of MFGM protein components on gut health and resistance to infections [17]. Additionally, clinical evidence studies have suggested that addition of bMFGM to infant formula during early infancy may promote beneficial outcomes in areas such as cognitive development, fat composition, cardiovascular risk markers, oral microbiota, and infection risk [18–22], thus potentially helping to narrow the gap in health outcomes between infants who receive human breast milk or infant formula. However, further research is needed to understand and confirm the efficacy, mechanism of action, and long-term benefits of adding bMFGM to infant formulas. In particular, the use of Event-Related Potential (ERP) provides a unique opportunity to assess bMFGM effects on neurocognitive development. During infancy, ERPs provides neural correlates of a variety of perceptual and cognitive functions and allows assessment of the infant’s ability to process stimuli that are critical for normal language acquisition at different ages [23, 24].

The Chilean infant Nutrition Trial (ChiNuT) is a randomized, double-blinded controlled trial that aims to assess addition of bMFGM to infant formula on physical growth, health, and brain development in the first year of life. Growth at 24 months of age (all participants) and Event-Related Potential (ERP) scores (participant subset) at baseline and 24 months of age will be the primary variables to compare infants who receive one of two study formulas: routine cow’s milk-based formula or a similar formula that has a source of bMFGM (whey protein-lipid concentrate, 5 g/L) through 12 months of age. A Human Milk reference group of participants receiving mother’s own milk will also be followed through 24 months of age. Secondary outcomes will include: micronutrient status, metabolic markers, complex lipids, body composition, and brain development outcomes such as sensory processing, memory, learning, language, and sleep-wake organization at different study time points through 24 months of age. Adverse health events were also collected through the study.

## Methods

### Study design

This study is a single center, double-blind (parent and researcher), and parallel randomized clinical trial, which is conducted under the umbrella of the Chilean Maternal and Infant Nutrition Observatory (ChiMINO). ChiMINO is a collaboration between the Institute of Nutrition and Food Technology (INTA) from the University of Chile, the Catholic University of Chile, and the South East Metropolitan Health Services of Santiago, Chile. This collaboration aims to provide observational and experimental evidence to achieve healthy maternal and infant growth and weight during the first 1000 days of life. The collaboration allows researchers from both academic institutions to have access to primary and secondary health centers from the South East Metropolitan Health Services of Santiago (SSMSO), in order to support recruitment and obtain secondary health data from the participants recruited into different study projects; in the SSMSO there were 9147 newborns in 2016 and at 3 months 3991 (63.3%) infants received exclusive breastfeeding [25, 26]. Breastfed infants were registered in a Human Milk (HM) Reference group and intent to consume mother’s own breast milk through 365 days of age. Infants receiving infant formula were randomized to receive one of two study formulas through 365 days of age (Table 1): 1) routine cow’s milk based infant formula (SF) or 2) or a similar formula with added whey protein-lipid concentrate (5 g/L, source of MFGM; Lacprodan® MFGM-10, Arla Foods Ingredients) (EF). Per 100 kcal, each study formula had docosahexaenoic acid (DHA) at 17 mg, arachidonic acid (ARA) at 25 mg, protein at 1.9 g, and iron at 1.2 mg; both study formulas also had a prebiotic blend of polydextrose (PDX) and galactooligosaccharides (GOS) (4 g/L) [27].

**Table 1:** Nutrient composition per 100 kcal (20 Calories/fl oz)

Nutrient	Study formula (target values)	
	SF	EF
Protein, g*	1.9	1.9
Fat, g†	5.3	5.3
Total Carbohydrate, g‡	11.4	11.4
Docosahexaenoic Acid, mg	17	17
Arachidonic Acid, mg	25	25
Vitamin A, IU	300	300
Vitamin D, IU	60	60
Vitamin E, IU	2	2
Vitamin K, mcg	9	9
Thiamin, mcg	80	80
Riboflavin, mcg	140	140
Vitamin B6, mcg	60	60
Vitamin B12, mcg	0.3	0.3
Niacin, mcg	1000	1000
Folic Acid, mcg	16	16
Pantothenic Acid, mcg	500	500
Biotin, mcg	3	3
Vitamin C, mg	12	12
Choline, mg	24	24
Inositol, mg	6	6
Calcium, mg	78	78
Phosphorus, mg	43	43
Magnesium, mg	8	8
Iron, mg	1.2	1.2
Zinc, mg	1	1
Manganese, mcg	15	15
Copper, mcg	75	75
Iodine, mcg	15	15
Selenium, mcg	2.8	2.5
Sodium, mg	27	27
Potassium, mg	108	108
Chloride, mg	63	63

* Sources of protein: skim milk and whey protein concentrate; INV-MFGM: Protein source includes bovine whey protein-lipid concentrate (5 g/L, source of MFGM)

† Sources of fat: base blend of palm olein, soybean, coconut, and high oleic sunflower oils; fungal-derived single cell oil (source of ARA); algal-derived single cell oil (source of DHA)

‡ Sources of carbohydrate: Available carbohydrate (source: lactose, galactose, glucose, fructose, epilactose): 10.8 g; Prebiotic oligosaccharides: 0.6 g, (source: prebiotic blend of polydextrose [PDX] and galactooligosaccharides [GOS; 1:1 ratio])

### Participants

Infants eligible for the HM Reference group were consuming mother’s own breast milk as the exclusive source of nutrition with the intent to feed mother’s own breast milk through approximately 180 days of age. Infants eligible for randomization to study formula were consuming infant formula as the sole source of nutrition for at least 48 h prior to randomization. Additional inclusion criteria for all infants were: 1) singleton birth; 2) up to 120 days of age at study registration or randomization; 3) birth weight between 2500 to 4500 g; 4) gestational age between 37 and 42 weeks; 5) history of normal growth (weight between and inclusive of the 10th and 90th percentiles on the WHO growth chart [28]) and 6) parents agreed not to enroll the infant in another interventional study and signed informed consent; inclusion criteria were asked by trained personnel on the recruitment telephonic call and then confirmed by trained dietitians based on the interview and primary care clinical history assessed on the first patient visit. Infant exclusion criteria included: use of complementary feeding; history of underlying endocrine, metabolic, or chronic diseases, congenital malformation, or any other condition that could interfere with the ability of the infant to ingest food, or to have normal growth and development; evidence of feeding difficulties or formula intolerance; immunodeficiency; and maternal illiteracy.

### Recruitment and randomization

Enrollment was planned for 540 participants in Santiago, Chile: 200 exclusively breastfed infants who were registered in the HM Reference group and 340 infants (whose mothers chose to initiate exclusive infant formula feeding before 4 months of age) who were randomized to either SF or EF formula group. Mothers were enrolled following one of two approaches: 1) In-person (through visits at the maternity ward of La Florida and Sótero del Rio Hospital or at primary health centers of the South East Metropolitan area of Santiago) in which study personnel invited mothers to participate in an infant growth study and registered their data on an electronic database or 2) Social media (urban area of Santiago), in which potential participants self-registered by adding their personal contact information in a database. Then, study personnel contacted registrants to conduct a short telephone interview to identify infants consuming formula or mother’s own breast milk. If the participant was currently consuming formula but not in an exclusive basis, a follow up call was scheduled until 4 months of age. Mother-infant pairs that appear to meet the inclusion criteria according to the telephone interview were invited to visit INTA where a trained dietitian verified inclusion and exclusion criteria, explained the study in detail, and obtained the written informed consent. Study registration or randomization, and the baseline evaluation was conducted at this visit. Randomization to study formula groups was conducted by using the permuted-block randomization method created at INTA. Group allocation was blinded to parents and study staff involved in data collection until all infants have completed their follow-up.

### Participant engagement

To ensure adequate retention of participants until 24 months, monthly calls will be conducted until 12 months and every 2 months, thereafter. Calls will check contact information, health status of the infant, potential adverse effect as well as informing about the course of the study and the next clinical visit.

### Ethical approval

This study is conducted in accordance with the ethical principles that have their origin in the Declaration of Helsinki and was consistent with Good Clinical Practice (GCP) and applicable regulatory requirements. The protocol and any amendments and the informed consent received approval from the Institutional Review Board of the Institute of Nutrition and Food Technology at the University of Chile. Written informed consent to participate was obtained from the parent or legal guardian of the participants during their baseline evaluation by trained dietitians. Signed informed consent was mandatory before proceeding with any of the study assessments.

### Protocol version and amendments

This study protocol is based on version 1.3 of the trial protocol which was reviewed on August 2018 by the ethics committee.

### Study outcomes

Growth was evaluated in all participants through 24 months of age. Three subsets of participants were additionally evaluated for body composition, metabolic and micronutrient status, and cognitive performance (Participant Subset 1); sleep patterns and auditory ERP (Participant Subset 2); language development (Subset 3). The primary outcomes of this study were: body weight, length and head circumference (all participants) and auditory ERP (Participant Subset 2). All data was collected in at least five study visits from registration/randomization until 24 months of age. Those visits are at baseline (before or at 120 days of age), 6 month of age (180 ± 14 days), 9 months of age (270 ± 14 days), 12 months of age (365 ± 14 days) and 24 months of age (730 ± 14 days). Additional visits were scheduled in participants that belong to the Subset 3 at 8 months of age (240 days ±14 days). Adverse events and compliance were followed up during the whole period of the study through monthly phone calls (Table 2).

**Table 2:** Measurements and visits conducted in ChiNuT

Study Outcome	Method/Samples	Randomization	Close-Out				
		≤ 120	180*	240*	270*	365*	730*
*ENROLLMENT:*							
Eligibility Screen		X					
Informed Consent		X					
Randomization		X					
*STUDY GROUPS:*							
*Standard formula (SF)*		X	X	X	X	X	X
*Experimental formula (EF)*		X	X	X	X	X	X
*Human milk Reference (HM)*		X	X	X	X	X	X
*ASSESSMENTS:*							
*Parent/Family*							
Sociodemographics	Questionnaire	X					
Family History	Questionnaire	X					
Maternal obstetric history	Questionnaire/Clinical Record	X					
Parental Height	Stadiometer	X					
Parental Weight	SECA Scale	X	X			X	X
Parental Waist Circumference	Circumference Tape	X	X			X	X
*All participants*							
APGAR score	APGAR test	X					
Clinical history	Clinical record	X	X			X	X
Length/Height	Stadiometer	X	X		X	X	X
Weight	SECA scale	X	X		X	X	X
Head Circumference	Circumference Tape	X	X		X	X	X
Mid Upper Arm Circumference	Circumference Tape	X	X		X	X	X
Dietary Assessment	24-Hr Recall Questionnaire		X		X	X	X
Medically confirmed AEs	Phone calls						
Protocol compliance	Phone calls						
*Participant Subset 1*							
Body composition	PeaPod®	X	X				
	Deuterium Dilution					X	X
Micronutrient Status	Fasting venous sample	X	X			X	X
Metabolic Markers	Fasting venous sample	X	X			X	X
Serum Phospholipids	Fasting venous sample	X	X				
Cognitive performance	Bayley-III, Cognitive Scale					X	
*Participant Subset 2*							
Sleep Pattern	Actigraphy	X				X	X
	BISQ	X				X	X
ERP	Dense array Electroencephalography	X					X
*Participant Subset 3*	Native Phoneme		X			X	
	Word Discovery			X			
	Rule Inference			X			
	CDI					X	X

*All time points ± 14 days

#### Anthropometrics

Parental height and weight were obtained from both participant’s parent(s) when possible with adequate equipment at Baseline and Day 180, 365, and 730. Participant birth weight and birth length (and other anthropometric information collected from birth until study registration or randomization) were obtained from the participant’s electronic health records due to the collaboration through the ChiMINO. Weight, recumbent length, mid-upper arm circumference, and head circumference were obtained by trained INTA nurses using standardized INTA protocols and with adequate equipment (SECA balance, SECA tape, SECA stadiometer) with a precision of 5 g (weight) and 0.1 cm (length and head circumference). Z-scores was estimated based on World Health Organization (WHO) growth standards [28].

#### Dietary assessment: early feeding questionnaire and 24-h recalls

During the first 9 months of life we collected dietary information based on an early feeding questionnaire that captured type of feeding (artificial, breastmilk, mixed), frequency, mode of preparation (in the case of artificial and mixed feeding), date of stop of exclusive and partial breastfeeding, date of initiation of complementary feeding including a list of 30 foods and beverages commonly consumed by Chilean infants based on previous studies of our group. At the 6-months visits all participating families were provided with standardized plates and spoons to aid in obtaining dietary intake information. The parent/caregiver was asked to recall the 24 h prior to the study visit and record infant formula intakes as well as complementary food intakes at Days 180, 270, 365, and 730. Dietary composition will be analyzed using Chilean Nutrient Databases complemented with International Nutrient Databases.

#### Body composition

Body composition was measured (Participant Subset 1) at Baseline and Day 180 by Airway displacement plethysmography (PeaPod®, Cosmed; designed for use in infants up to 6 months of age or infants weighing up to 10 kg), which is a well-established, reliable system that assesses body composition by whole-body densitometry using two-compartment model [29]. During the evaluation period, the system was used in a temperature-controlled room and routinely calibrated once a day. Infants were evaluated undressed in the test chamber for 3 min. Body volume is measured by air displacement plethysmography, body weight is measured using an electronic scale, and body composition is calculated from these data using the “Fomon” model [30]. Fat mass (FM) density is assumed to be constant (0.9007 g/mL), whereas free fat mass (FFM) density varies from 1.063 g/mL at birth to 1.067 g/m at age of 6 months [30]. FM is calculated as (weight x BF%) and FFM as (weight-FM) [31].

At Day 365 and 730, deuterium (D_2_O) dilution (DD) [32] was used because infants would exceed the weight allowed on the PeaPod®. D_2_O is a non-radioactive isotope used in this technique to determine total body water (TBW), which allows the estimation of body fat and FFM. For each participant, an initial saliva sample (~ 2 mL) was collected: a cotton wool swab was rolled in the infant’s mouth for approximately 2 minutes and then placed into a syringe barrel, and compressed against the head of the syringe to extract saliva. The process can be repeated if necessary to meet the amount of saliva needed (~ 2 mL); samples will be centrifuged immediately (3000 rpm × 3 min). A dose of 99.8% D_2_O (1 mg in 1 mL of sterile water, assuming an average weight of 9 kg at 365 days and 12.5 at 730 days, to produce an enrichment ~ 75–200 ppm; Cambridge Isotopes Laboratory, USA) was administered orally. One hour after the dose participants received 1 or 2 biscuits and a cold 100 mL drink. After 3 hours, the second saliva sample was obtained similarly. All deuterated samples will be stored at − 20 °C until analyzed by continuous flow isotope ratio mass spectrometry (Sercon Group, Crewe, UK) at the INTA Stable Isotopes Laboratory from, University of Chile. TBW will be calculated according the following equation [33]:

$$ \left(\mathrm{T}\mathrm{BW}\right)\ \left(\mathrm{kg}\right)=\left[\left(\left(\mathrm{T}\ \mathrm{x}\ \mathrm{A}/\mathrm{a}\right)\ \mathrm{x}\left(\left(\mathrm{Ea}-\mathrm{Et}\right)/\left(\mathrm{Es}-\mathrm{Ep}\right)\right)\right)/1000\right]/1.04 $$

Where A is the amount of dose solution drunk (g); a, amount of dose solution diluted in T (g); T, amount of water in which “a” was diluted in (g); Ea, enrichment of diluted dose; Et, enrichment of water used to dilute the dose; Ep, enrichment of baseline simple; Es, enrichment of post-dose sample; 1.04, correction factor for overestimation of TBW by the use of D_2_O.

#### Blood measures

At Baseline and Day 180, 365, and 730 (Participant Subset 1) a total of approximately 7 mL of whole blood was collected in fasting state (two to 4 hours fasting) via venipuncture. If the attempt to obtain a 7 mL blood sample is unsuccessful, the participant may continue consuming study formula and return within 7 days or less for another attempt to obtain the 7 mL blood sample. Tubes will be centrifuged within 4 h of collection and plasma and serum aliquots will be stored at − 80 °C until analysis. Insulin, adiponectin, and leptin will be measured by radioimmunoassay (RIA); glycaemia, lipids and high-sensitivity C-reactive protein (HS CRP) analysis will be conducted using standard procedures at the Nutrition Laboratory of Catholic University. Total Insulin-like Growth Factor-1 (IGF-1) levels will be determined by RIA [34] at the Laboratory of the Institute of Maternal and Child Research (IDIMI), University of Chile. Serum Zinc will be measured following flame atomic absorption spectrometry (Perkin Elmer, Model 2280) serum ferritin will be measured by sandwich Enzyme-linked Immunosorbent Assay (ELISA) [35], transferrin receptors by ELISA (Ramco Laboratories, Texas, USA); and serum iron by graphite furnace atomic absorption spectrometry (Simaa 6100, Perkin Elmer) at the Micronutrient Laboratory at INTA, University of Chile. Sphingomyelin (SM) and gangliosides (GS) will be analyzed by lipid extraction and High Performance Liquid Chromatography (HPCL) spectrometry (Avanti Polar Lipids).

#### Bayley scales of infant and toddler development

The Bayley Scales have been used primarily as a pediatric screening instrument to identify delayed development, but has also become widely used and accepted as a comparative measure of mental development in research studies. The current 3rd edition (Bayley-III) [36] is used to evaluate infants and children from 1 to 42 months of age in five developmental domains: cognitive, motor, language (administered with the child by trained examiners), social-emotional and adaptive behavior (administered via parent questionnaires) [37]. The Bayley-III is suited to administration by multidisciplinary teams of professionals. It was initially standardized in a US population, but has been previously translated and adapted for use in Spanish [38] and used in Chilean pediatric populations. Domain subtests can be administered individually; in the present study we have elected to administer the cognitive scale at Day 365 (Participant Subset 1). All measurements were conducted by phycologist with a master’s degree and certification to conduct Bayley measurements.

#### Event-related potential (ERP)

ERPs were collected at Baseline and Day 730 (Participant Subset 2), using a geodesic sensor net (128 scalp sites; Electric Geodesic, Inc., Eugene, Oregon, USA) to collect electroencephalographic (EEG) recordings. The vertex was used as the on-line reference electrode. The signal was sampled at 1000 Hz and bandpass filtered on-line at 0.1 to 100 Hz.

Infants were seated on their parents’ laps in a comfortable chair that is positioned with its center equidistant from the face of two speakers that are attached to opposite walls inside a sound-attenuated and electrically shielded room. An experimenter engaged the children’s attention with a silent puppet show or toys. The stimuli was consonant–vowel syllables: the standard stimulus is a Consonant-vowel (CV) syllable phonetically relevant in Spanish (ta) and two CV syllables were used as deviants: a native deviant (da) and a non-native deviant (sha). Stimuli were presented in an oddball paradigm that contained a standard syllable (80%), a native deviant syllable (10%) and a non-native deviant syllable (10%) for a total of 1000 stimuli. The stimulus onset-to-onset interval is 930 ms. All stimuli (E-Prime software, Psychology Software Tools, Inc.) were amplified to a calibrated level of 60 dB sound pressure level (SPL). Sounds were in free-field via left and right speakers.

After recording, stimulus triggers will be exported (Net Station) and analyzed (BESA). The signals will be re-referenced off-line to an average (whole head) reference and bandpass filtered at 0.1–10 Hz will be used. The continuous EEG will be segmented into epochs according to the stimulus type (native deviant, non-native deviant and standard), with the segment length being the same as each onset-to-onset inter trial stimulus. In addition, a 50-ms pre-stimulus segment will be included for baseline correction. Segments of data with excessive movement artifact will be rejected by visual inspection, and noisy channels will be identified and rejected. A channel rejection threshold is set at < 20% and for a second analysis steps, rejected channels will interpolate using a spherical method.

For ERP averaging, continuous data will filter with a 1–15 Hz bandpass filter and epoch -300 to 1000 ms around stimulus presentation (i.e. “time 0”). An artifact rejection criterion of ±500 μV will be used to reject noisy epochs and a threshold of maximum percent rejected will be set at < 30%.

At baseline, we expect to find differences between the 3 types of stimuli. The difference will be reflected in the amplitude and latency of the different waves obtained. For deviant stimuli we expect greater amplitude and longer latency.

At Day 730 experiment was repeated using the same settings. There were changes in the response according to maturation of the central nervous system. The changes were produced for the maturation of cognitive abilities as language discrimination and attention. The language development will be reflect by responses with smaller amplitudes and shorter latencies. Besides, the differences among stimuli will decrease due to learning the native language.

#### Actigraphy

Actigraphy was obtained at Baseline, Day 365, and Day 730 (Participant Subset 2). Actigraphs are devices which record motor activity and allow to study the sleep-wake cycle. Its use has several advantages as being non-invasive, very light, and gives the possibility to be used continuously for prolonged periods of time while following their usual lifestyle at home. The actigraph has an internal memory, an accelerometer (sensitivity < 0.01 g), and records, digitalizes, and stores movement units for each successive 1 min interval.

Actigraphs were be installed on the infant’s left leg, 1 week before coming to the laboratory for the ERP study at baseline, and were installed at home at 365 and 730 days. Parents were asked not to remove the device during a week even during bathing. Recordings were not performed if the infant was sick.

Data will be processed on a min-by-min basis with a locally developed automated software. To avoid inaccurate identification of short sleep and wake episodes which are a main source of errors and controversy [39–41], we reassessed this first detection of sleep and wake episodes, generating a new sequence of sleep and wake episodes with durations lasting at least 5 min. Shorter changes were included in the ongoing episode. For example, the sequence SSSSSSSSWWSWSSSS (S = sleep, W = wake) based on 1-min length will become SSSSSSSSSSSSSS. Each 24-h cycle will be divided into nocturnal and diurnal periods: the nocturnal period begins with the onset of the first sleep episode after 8:00 pm that continues for at least 30 min. The diurnal period begins with the first wake episode after 6:00 am that continues for at least 30 min.

The following sleep-wake parameters will be calculated:

1. Nocturnal period (time spent between nighttime sleep onset and wake-up the next morning):
  total sleep time (TST), time from sleep onset to the end of the final sleep episode minus time spent awake
  wakefulness after sleep onset (WASO): the time spent awake between sleep onset and end of sleep
  nighttime awakenings (NA): number of wakefulness episodes within the nocturnal period
2. Diurnal period (time spent between wake-up and nighttime sleep onset):
  number of naps (N)
  total daytime sleep (TDS): time from waking onset to the end of the final waking episode minus time spent asleep

#### Brief infant sleep questionnaire (BISQ)

A Spanish and standardized version of the BISQ [42], a sleep questionnaire assessing the infant’s typical sleep patterns based on parental reports, will be used at Baseline, Day 365, and Day 730 (Participant Subset 2). The questionnaire has been validated against sleep diary and actigraphic measures; and the derived measures will be: sleep onset time; nocturnal sleep duration; daytime sleep duration; number of night awakenings; and sleep position (supine, side and prone) [43]. Parents were instructed to fill out the BISQ during the same 7-day period that the participant wears the actigraph device.

#### Language acquisition

Early language acquisition in healthy infants is characterized by distinct milestones during the first year of life [44, 45]. Among these milestones, native phoneme repertory, word learning from continuous speech, rule inference and communicative development were evaluated (Participant Subset 3).

1. Native Phoneme Repertory

We will measure the neural basis of a hallmark of early linguistic development, which is the learning of the native phoneme repertory [46–48]. Before 10 months of age, healthy infants distinguish native and non-native phonemes. Near 12 months of age, healthy infants reduce sensitivity to nonnative contrasts and increase sensitivity to native contrasts as a product of neural maturation and experience with native language. To measure phoneme discrimination for native and non-native phonemes, a synthetic consonant-vowel stimuli was prepared [46] to study categorical perception in English and Hindi speakers. This continuum comprises 16 steps along the voiced place-of-articulation dimension from the bilabial /b/ to the dental /d/ and retroflex /D/, associated with the vowel /a/ (hereafter S1 to S16). Along this continuum, adult native Spanish speakers perceive two phonetic categories (S1–S6 as /pa/ and the following as /ta/) whereas adult native Hindi speakers perceive three (S1–S6 as /ba/, S7–S10 as /da/, and S11–S16 as retroflex /Da/). Crucially, infants at 9 months of age raised in a native Spanish environment respond equally well to both boundaries, at 12 to 14 months of age infants react similar to Spanish-speaking adults reducing their neural response to the Hindi boundary [48], which reveals early aspects of the native phoneme repertory learning. To measure that change in the response across the first year, we will use the Mismatch response, a classic electrophysiological paradigm directed to evaluate the response for native and non-native phonemes in young infants. EEG recordings of the brain activity associated to detect a mismatch of phonemes /pa/, /ta/ and Tha/ before and after the repertory of native phonemes has been defined for consonants [48–50].

2) Word learning from continuous speech

The ability of infants to segment fluent speech into potential words and to memorize those words for later recognition [51, 52] were captured by recording EEG activity [53]. After 3 min of familiarization with a continuous speech stream containing 4 non-sense tri-syllabic words presented at a fixed frequency, EEG activity were measured for 3 min. Using a frequency tagging procedure, processing of the syllables and the discovery of the tri-syllabic nonsense word were measured.

3) Rule Inference

Rule inference is a learning task where infants at 8 months of age should infer that an auditory cue predicts the occurrence, a second later, of a visual event. We thus measured the inferential learning by using a remote, free-head eye tracking technique, which allows to record the infant behavior by analyzing the position of their gaze every 20 ms over the target [54].

4) MacArthur Communicative Development Inventory (CDI)

The CDI [55] is an assessment tool to measure infant language development. Age-appropriate forms of the CDI can be administered at 12 and 18 months owing to changes in the nature of language. The progress of the size of the comprehensive and productive vocabulary will be evaluated using the Spanish adapted CDI version [56].

### Sample size

Based on preliminary data collected in a pilot study comparing children from the same baseline population who had received infant formula or breastfeeding [57, 58], to detect a difference of: 0.8 kg weight (mean 12.5 ± 1.6), 0.8 kg/m^2^ body mass index (BMI) (17.5 ± 1.5) and 1.5 cm length (86.3 ± 3.0) at 730 days of age (two-tail, α = 0.05, 80% power) we needed 120 infants per group. We estimate a 30% of loss to follow-up over a 2 year period; thus, 170 infants per group were enrolled for the general follow-up and the anthropometric assessments. Anthropometric and general assessment, were carried out for the entire sample whereas body composition, metabolic/micronutrient assessment, cognitive studies, and language development were only carried out in participant subsets. For the body composition and metabolic/micronutrient assessments a random sample of 80 infants per group were randomly selected from the entire sample. Allowing for a 50% loss to follow-up this sample size should allow us to detect at 24 months of age differences of: 0.3 kg of fat mass (3.0 kg mean ± 0.5); 1.6 mIU/mL insulin (mean 4.2 mIU/mL ±2.5) (based on preliminary data collected in a pilot study comparing formula-fed and breastfed children from the same baseline population) [58, 59].

For neurocognitive outcomes, we needed 25 infants per group to detect differences of − 4.0 μV ERP component amplitude (mean − 5.21 ± 6.23) at 24 months of age (two-tail, α = 0.05, 80% power) [60]. Allowing for a 50% of loss to follow up; thus, final sample sizes will be 50 infants per group.

### Statistical methods

Anthropometric results will be expressed as Z scores according to the growth standards of the World Health Organization (WHO) [61]. Variables will be described using averages and standard deviations, or medians and interquartile range (normal or non-normal variables, respectively).

To compare response variables (growth, body composition, and metabolic markers and micronutrients) among groups (HM Reference, SF, EF) cross-sectionally (at Days 180, 365, and 730 days) Pearson’s Chi square and Fisher exact test will be used for categorical data and t-test for continuous data; linear and logistic multiple regressions will be performed to assess group differences (SF versus EF, SF versus HM, EF versus HM) adjusting for potential confounders such as sex, educational level of mothers, weight gain during pregnancy, smoking during pregnancy, etc. (eg allowing for ineffective randomization).

To evaluate the effect of feeding mode on changes in growth, body composition, metabolic markers and micronutrients between baseline and the 180 and 365 days of age visits, baseline information for each of the outcomes will be included in the multiple linear and logistic regression. Finally, to assess the overall effect of the mode of feeding on growth, metabolic markers and micronutrient trajectories mixed linear regression models (mixed models), adjusting for potential confounders will be performed. Cases without information on the response variable (at Days 180 and 365) will be excluded from all analyses. Statistical analyses will be performed both 1) according to the original allocation independent of degree of adherence, and 2) based on feeding compliance up to 180 days of age: In study formula groups, noncompliance will be defined as feeding of non-study formula or breastfeeding for > 10% of feedings (or > 3 bottles/week) over ≥1 week. In the HM Reference group, participants will be primarily receiving mother’s own milk during their first 6 months of age (< 10% of feedings or < 3 bottles of formula/week).

Statistical analysis will be done in SAS version 9.2 (SAS Institute Inc., Cary, NC) and Stata version 12.0 (StataCorp LP, College Station, TX); *p* values < 0.05 will be considered significant.

### Safety monitoring

Participants were contacted monthly to check the presence of a number of symptoms, starting date, duration, need of treatment (including the need of hospitalization), medical diagnoses (if applicable) and resolution. Adverse effects were informed to the ethics committee and to the internal safety monitoring committee of the study; aggregated reports of adverse effects were examined every 4 months.

## Discussion

Even though exclusive breastfeeding until 6 months of age is the recommendation by the World Health Organization to achieve optimal growth and cognitive development [5, 62] only a 40% of infants in the world are currently following such recommendation [63]. Multiple reasons are reported explaining this low percentage and some of them correspond to health conditions that are not necessarily reversible. Thus, there is a need in pediatric nutrition to develop infant formula that could supply similar functional nutrients as the ones found in mother’s own breast milk in order to narrow the gap in neurodevelopmental outcomes.

Addition of bMFGM to infant formula has already demonstrated potential beneficial effects with respect to neurodevelopment, infectious diseases, and cholesterol metabolism; however human studies have been scarce and have used different MFGM fractions limiting the generalizability of the findings [12] . The present study expects to contribute to the discussion conducting a well-powered RCT that will include ERPs, and sleep-wake patterns to characterize neurocognitive development, as well as standard measures of growth, body composition, metabolic and micronutrients status.

This study was conducted in a controlled, double-blinded and longitudinal setting, which reduced possible selection and information bias. Additionally, the study was conducted in a larger study population when comparing with previous similar studies [18–22], which may increase the robustness of the results obtained. Moreover, the study population was very similar in terms of socioeconomic status, which may also reduce the risk of bias in the study.

A limitation could be related to adherence because we will be unable to objectively assess whether the participant is exclusively consuming study formula or exclusively consuming mother’s own breast milk during the first 6 months as the protocol stands. However, monthly follow up calls were conducted in order to assess whether the mothers are complying and stimulate formula consumption or breastfeeding.

## Data Availability

The data collected during this study are not publicly available yet since is in a cleaning stage. However the data are available from the corresponding author on reasonable request.

## Abbreviations

MFGM: Milk fat globule membranes
bMFGM: Bovine source of milk fat globule membranes
DHA: Docosahexaenoic
ARA: Arachidonic acid
SF: Standard formula
EF: Experimental formula
ChINuT: Chilean infant nutrition trial
ERP: Event-related potential
ChiMINO: Chilean maternal and infant nutrition observatory
INTA: Institute of nutrition and food technology
HM: Human milk
PDX: Polydextrose
GOS: Galactooligosaccharides
GCP: Good clinical practice
WHO: World health organization
FM: Fat mass
FFM: Free fat mass
DD: Deuterium (D_2_O) dilution
TBW: Total body water
IDIMI: Laboratory of the institute of maternal and child research
EEG: Electroencephalography
CV: Consonant-vowel
BMI: Body mass index
HS CRP: High-sensitivity C-reactive protein
IGF-1: Insulin-like growth factor-1
ELISA: Enzyme-linked immunosorbent assay
HPCL: High performance liquid chromatography

## References

[1] Ben-Shlomo Y, Kuh D (2002). A life course approach to chronic disease epidemiology: conceptual models, empirical challenges and interdisciplinary perspectives. Int J Epidemiol.

[2] Uauy R, Kain J, Mericq V, Rojas J, Corvalán C (2008). Nutrition, child growth, and chronic disease prevention. Ann Med.

[3] Uauy R, Kain J, Corvalan C (2011). How can the developmental origins of health and disease (DOHaD) hypothesis contribute to improving health in developing countries?. Am J Clin Nutr.

[4] de Onis M, Garza C, Onyango AW, Rolland-Cachera MF (2009). Pédiatrie lCdndlSfd: [WHO growth standards for infants and young children]. Arch Pediatr.

[5] World Health Organization UNICEF. In: WLC-i-P D, editor. Global strategy for infant and young child feeding. Geneva: World Health Organization; 2003.

[6] Binns C, Lee M, Low WY (2016). The long-term public health benefits of breastfeeding. Asia Pac J Public Health.

[7] Committee on Nutrition: Iron fortification of infant formulas (1999). American academy of pediatrics. committee on nutrition. Pediatrics.

[8] Raiten DJ, Talbot JM, Waters JH, Life Sciences Research Office (1998). Assessment of nutrient requirements of infant formulas.

[9] SCF (2003). Report of the Scientific Committee on Food on the Revision of Essential Requirements of Infant Formulae and Follow-on Formulae (adopted on 4 April 2003).

[10] European Commission: Infant formulae and follow-on formulae and amending Directive 1999/21/EC. In*.*, vol. Regulation (EC) No 2006/141; 2006.

[11] Martin CR, Ling PR, Blackburn GL. Review of infant feeding: key features of breast milk and infant formula. Nutrients. 2016;8(5):279–89. 10.3390/nu8050279 PMC4882692 27187450

[12] Timby N, Domellöf M, Lönnerdal B, Hernell O (2017). Supplementation of infant formula with bovine Milk fat globule membranes. Adv Nutr.

[13] Oshida K, Shimizu T, Takase M, Tamura Y, Shimizu T, Yamashiro Y (2003). Effects of dietary sphingomyelin on central nervous system myelination in developing rats. Pediatr Res.

[14] Palmano K, Rowan A, Guillermo R, Guan J, McJarrow P (2015). The role of gangliosides in neurodevelopment. Nutrients.

[15] Wang B, Yu B, Karim M, Hu H, Sun Y, McGreevy P, Petocz P, Held S, Brand-Miller J (2007). Dietary sialic acid supplementation improves learning and memory in piglets. Am J Clin Nutr.

[16] Liu H, Radlowski EC, Conrad MS, Li Y, Dilger RN, Johnson RW (2014). Early supplementation of phospholipids and gangliosides affects brain and cognitive development in neonatal piglets. J Nutr.

[17] Ten Bruggencate SJ, Frederiksen PD, Pedersen SM, Floris-Vollenbroek EG, De Bos EL, Van Hoffen E, Wejse PL (2016). Dietary milk-fat-globule membrane affects resistance to diarrheagenic *Escherichia coli* in healthy adults in a randomized, placebo-controlled, double-blind study. J Nutr.

[18] Timby N, Lonnerdal B, Hernell O, Domellof M (2014). Cardiovascular risk markers until 12 mo of age in infants fed a formula supplemented with bovine milk fat globule membranes. Pediatr Res.

[19] Timby N, Domellof E, Hernell O, Lonnerdal B, Domellof M (2014). Neurodevelopment, nutrition, and growth until 12 mo of age in infants fed a low-energy, low-protein formula supplemented with bovine milk fat globule membranes: a randomized controlled trial. Am J Clin Nutr.

[20] Timby N, Hernell O, Vaarala O, Melin M, Lonnerdal B, Domellof M (2015). Infections in infants fed formula supplemented with bovine milk fat globule membranes. J Pediatr Gastroenterol Nutr.

[21] Rosqvist F, Smedman A, Lindmark-Månsson H, Paulsson M, Petrus P, Straniero S, Rudling M, Dahlman I, Risérus U (2015). Potential role of milk fat globule membrane in modulating plasma lipoproteins, gene expression, and cholesterol metabolism in humans: a randomized study. Am J Clin Nutr.

[22] Billeaud C, Puccio G, Saliba E, Guillois B, Vaysse C, Pecquet S, Steenhout P (2014). Safety and tolerance evaluation of milk fat globule membrane-enriched infant formulas: a randomized controlled multicenter non-inferiority trial in healthy term infants. Clin Med Insights Pediatr.

[23] Choudhury N, Benasich AA (2011). Maturation of auditory evoked potentials from 6 to 48 months: prediction to 3 and 4 year language and cognitive abilities. Clin Neurophysiol : Official J Int Fed Clin Neurophysiol.

[24] Riva V, Cantiani C, Benasich AA, Molteni M, Piazza C, Giorda R, Dionne G, Marino C (2017). From CNTNAP2 to early expressive language in infancy: the mediation role of rapid auditory processing. Cereb Cortex.

[25] Ministry of Health. Vigilancia del Estado Nutricional de la población bajo control y de la lactancia materna en el Sistema Público [Surveillance of the Nutritional Status of the population under control and breastfeeding in the Public Health System of Chile]. Chile: Public Health Office; 2016.

[26] Department of Statistics and Health Information (DEIS). Nacimientos inscritos según edad de la madre por región y comuna de residencia de la madre [ Registered births according to age of the mother by region and commune of residence of the mother]. Chile: Ministry of Health; 2016.

[27] Brenna JT, Varamini B, Jensen RG, Diersen-Schade DA, Boettcher JA, Arterburn LM (2007). Docosahexaenoic and arachidonic acid concentrations in human breast milk worldwide. Am J Clin Nutr.

[28] Organization WH (2009). WHO child growth standards and the identification of severe acute malnutrition in infants and children.

[29] Ellis KJ, Yao M, Shypailo RJ, Urlando A, Wong WW, Heird WC (2007). Body-composition assessment in infancy: air-displacement plethysmography compared with a reference 4-compartment model. Am J Clin Nutr.

[30] Fomon SJ, Haschke F, Ziegler EE, Nelson SE (1982). Body composition of reference children from birth to age 10 years. Am J Clin Nutr.

[31] Urlando A, Dempster P, Aitkens S (2003). A new air displacement plethysmograph for the measurement of body composition in infants. Pediatr Res.

[32] Devakumar D, Grijalva-Eternod CS, Roberts S, Chaube SS, Saville NM, Manandhar DS, Costello A, Osrin D, Wells JC (2015). Body composition in Nepalese children using isotope dilution: the production of ethnic-specific calibration equations and an exploration of methodological issues. Peer J.

[33] Tam N, Nolte HW, Noakes TD (2011). Changes in total body water content during running races of 21.1 km and 56 km in athletes drinking ad libitum. Clin J Sport Med.

[34] Iniguez G, Ong K, Bazaes R, Avila A, Salazar T, Dunger D, Mericq V (2006). Longitudinal changes in insulin-like growth factor-I, insulin sensitivity, and secretion from birth to age three years in small-for-gestational-age children. J Clin Endocrinol Metab.

[35] Conradie JD, Mbhele BE (1980). Quantitation of serum ferritin by enzyme-linked immunosorbent assay (ELISA). S Afr Med J.

[36] Albers CA, Grieve AJ: Test Review: Bayley, N. (2006**).** Bayley scales of infant and toddler development– third edition. San Antonio, TX: Harcourt assessment. J Psychoeduc Assess 2007, 25(2):180–190.

[37] Bode MM, D'Eugenio DB, Mettelman BB, Gross SJ (2014). Predictive validity of the Bayley, third edition at 2 years for intelligence quotient at 4 years in preterm infants. J Dev Behav Pediatr.

[38] Farkas C. Manual de administraciοn version en espanol del BAYLEY-III. Proyecto FONDECYT No 1060778. 2007.

[39] Insana SP, Gozal D, Montgomery-Downs HE (2010). Invalidity of one actigraphy brand for identifying sleep and wake among infants. Sleep Med.

[40] Meltzer LJ, Walsh CM, Traylor J, Westin AM (2012). Direct comparison of two new actigraphs and polysomnography in children and adolescents. Sleep.

[41] So K, Adamson TM, Horne RS (2007). The use of actigraphy for assessment of the development of sleep/wake patterns in infants during the first 12 months of life. J Sleep Res.

[42] Ministerio de Sanidad Política Social e Innovación: BISQ (Brief Infant Sleep Questionnaire), breve cuestionario del sueño Adaptado de Sadeh, A, 2004. In Guía de Práctica Clínica sobre Trastornos del Sueño en la Infancia y Adolescencia en Atención Primaria. Edited by Ministerio de Ciencia e Innovación. Madrid: 2011.

[43] Sadeh A (2004). A brief screening questionnaire for infant sleep problems: validation and findings for an internet sample. Pediatrics.

[44] Kuhl PK (2004). Early language acquisition: cracking the speech code. Nat Rev Neurosci.

[45] Gervain J, Mehler J (2010). Speech perception and language acquisition in the first year of life. Annu Rev Psychol.

[46] Werker JF, Lalonde CE (1988). Cross-language speech perception: initial capabilities and developmental change. Dev Psychol.

[47] Dehaene-Lambertz G, Pena M (2001). Electrophysiological evidence for automatic phonetic processing in neonates. Neuroreport.

[48] Pena M, Werker JF, Dehaene-Lambertz G (2012). Earlier speech exposure does not accelerate speech acquisition. J Neurosci.

[49] Kuhl PK, Williams KA, Lacerda F, Stevens KN, Lindblom B (1992). Linguistic experience alters phonetic perception in infants by 6 months of age. Science.

[50] Werker JF, Cohen LB, Lloyd VL, Casasola M, Stager CL (1998). Acquisition of word-object associations by 14-month-old infants. Dev Psychol.

[51] Christophe A, Mehler J, Sebastián-Gallés N (2001). Perception of prosodic boundary correlates by newborn infants. Infancy.

[52] Saffran JR, Aslin RN, Newport EL (1996). Statistical learning by 8-month-old infants. Science.

[53] Kabdebon C, Pena M, Buiatti M, Dehaene-Lambertz G (2015). Electrophysiological evidence of statistical learning of long-distance dependencies in 8-month-old preterm and full-term infants. Brain Lang.

[54] Gervain J, Macagno F, Cogoi S, Pena M, Mehler J (2008). The neonate brain detects speech structure. Proc Natl Acad Sci U S A.

[55] Heilmann J, Weismer SE, Evans J, Hollar C (2005). Utility of the MacArthur—bates communicative development inventory in identifying language abilities of late-talking and typically developing toddlers. Am J Speech-Lang Pathol.

[56] Farkas C. Inventario del Desarrollo de Habilidades Comunicativas McArthur-Bates (CDI): propuesta de una versión abreviada. Universitas Psychol. 2011;10(1):245–62.

[57] Quinn PC, Doran MM, Reiss JE, Hoffman JE (2010). Neural markers of subordinate-level categorization in 6- to 7-month-old infants. Dev Sci.

[58] Corvalán C: Estudio de evaluación de impacto nutricional de la utilización de fórmulas sucedáneas de leche maternal (fórmula de inicio) versus leche purita fortificada en parámetros bioquímicos y composición corporal en una cohort de niños menores de 12 meses. Licitación ID: 757L183LLP14 In. Chile: Ministerio de Salud; 2014.

[59] Kain J, Corvalan C, Lera L, Galvan M, Uauy R (2009). Accelerated growth in early life and obesity in preschool Chilean children. Obesity (Silver Spring).

[60] Choudhury N, Benasich A (2010). Maturation of auditory evoked potentials from 6 to 48 months: prediction to 3 and 4 year language and cognitive abilities. Clin Neurophysiol.

[61] Child growth standards. WHO Anthro (versión 3.2.2, January 2011) and macros [http://www.who.int/childgrowth/software/en/].

[62] World Health Organization (2001). Report of the expert consultation of the optimal duration of exclusive breastfeeding, Geneva, Switzerland, 28–30 March 2001.

[63] UNICEF (2017). Tracking progress for breastfeeding policies and programmes: global breastfeeding scorecard 2017.

